# HELLP syndrome and associated factors among pregnant women with preeclampsia/eclampsia at a referral hospital in southwestern Uganda: a cross-sectional study

**DOI:** 10.1186/s12884-024-06835-y

**Published:** 2024-10-01

**Authors:** Fadumo Mohamed Abdullahi, Yarine Fajardo Tornes, Richard Migisha, Paul Kato Kalyebara, Leevan Tibaijuka, Joseph Ngonzi, Musa Kayondo, Onesmus Byamukama, Stuart Turanzomwe, Joseph Rwebazibwa, Brenda Ainomugisha, Rogers Kajabwangu, Godfrey R. Mugyenyi, Henry Mark Lugobe

**Affiliations:** 1 Department of Obstetrics & Gynecology, Mbarara University of Science and Technology, Mbarara, Uganda; 2 Department of Physiology, Mbarara University of Science and Technology, Mbarara, Uganda; 3 Department of Obstetrics and Gynecology, Mbarara Regional Referral Hospital, Mbarara, Uganda; 4 Division of Experimental Medicine and Immunotherapeutics, Department of Medicine, University of Cambridge, Cambridge, UK

**Keywords:** HELLP syndrome, Preeclampsia, Eclampsia, Uganda, Prevalence

## Abstract

**Background:**

Hemolysis Elevated Liver Enzymes Low Platelets (HELLP) syndrome, a complication of preeclampsia/eclampsia, is associated with severe maternal morbidity and mortality. In resource-limited settings, such as Uganda, gaps in routine laboratory assessments may lead to underdetection of HELLP syndrome. This study determined the prevalence and factors associated with HELLP syndrome among pregnant women with preeclampsia/eclampsia at Mbarara Regional Referral Hospital (MRRH), southwestern Uganda.

**Methods:**

A cross-sectional study was conducted at the high-risk ward of the MRRH from December 2022 to June 2023. Pregnant women diagnosed with preeclampsia or eclampsia were enrolled consecutively. Participants’ sociodemographic and clinical data were collected using an interviewer-administered questionnaire. The diagnosis of complete HELLP syndrome was made based on the Tennessee classification: aspartate aminotransferase enzyme ≥ 70 IU/L, platelet counts < 100,000 cells/µL, and serum lactate dehydrogenase enzyme ≥ 600 IU/L. We used multivariable modified Poisson regression analysis to determine factors associated with HELLP syndrome.

**Results:**

A total of 129 participants with a mean age of 28 ± 6.6 years were enrolled in the study. The prevalence of HELLP syndrome was 18.6% (*n* = 24; 95% CI: 12.7–26.3%). Independent factors associated with HELLP syndrome were maternal age (adjusted prevalence ratio [aPR]: 4.96; 95% CI: 1.57–15.65; for mothers aged < 20 years compared to those aged 20–34 years), the presence of epigastric pain (aPR: 5.89; 95% CI: 1.41–14.63), and referral from other health facilities (aPR: 3.14; 95% CI: 1.27–7.72).

**Conclusion:**

Approximately 2 of the 10 women who presented with preeclampsia or eclampsia had HELLP syndrome. It is more common among teenage mothers, those with a history of epigastric pain and those referred from lower health facilities. Incorporating routine laboratory testing for HELLP syndrome in the diagnostic protocol for preeclampsia or eclampsia, especially among adolescent mothers, those experiencing epigastric pain, and those referred from lower health facilities, could enhance timely detection and management of mothers with preeclampsia whose pregnancies are complicated by HELLP syndrome.

## Background

Hemolysis Elevated Liver Enzymes, Low Platelet count (HELLP), is an obstetric complication, often regarded as a distinct form of preeclampsia [1]. The prevalence of HELLP syndrome among women with preeclampsia/eclampsia has been shown to vary with 4.9% in Iran [2], 9.1% in Zimbabwe [3], 13% in Ethiopia [4] and 40% in India [5]. Several factors, including maternal age, gestational age, parity, multiple pregnancy, previous hypertension, diabetes mellitus, obesity and the presence of chronic hepatic conditions, have been reported to increase the risk of HELLP syndrome in women who have preeclampsia/eclampsia [6, 7]. The occurrence of preeclampsia and eclampsia demonstrates significant regional variation, with estimated incidence rates ranging from 1.6 to 10 per 10,000 deliveries in developed nations, contrasting with rates of 50 to 151 per 10,000 deliveries in developing countries [8]. Data on occurrence of preeclampsia/eclampsia in Uganda are limited; however, one study in Northern Uganda reported preeclampsia incidence of 4.3% [9].

Several complications of HELLP syndrome have been reported in patients with preeclampsia or eclampsia, including acute respiratory failure, severe kidney injury, hepatic damage and liver rupture, placental abruption, blood transfusion and coagulation disorders [10, 11].

Given the high risk of unfavorable maternal and perinatal outcomes among women who have had preeclampsia/eclampsia and HELLP syndrome, it is critical to identify women in our setting who are likely to have HELLP syndrome to plan tailored interventions. This study determined the prevalence and associated factors of HELLP syndrome among women with preeclampsia and eclampsia at a tertiary care hospital in southwestern Uganda.

## Methods

### Study design and setting

This was a cross-sectional study conducted on the high-risk ward of Mbarara Regional Referral Hospital (MRRH) from December 2022 to June 2023. The MRRH is a tertiary care facility located in southwestern Uganda, Mbarara city. The hospital serves as a regional referral hospital for southwestern Uganda and as a teaching hospital for Mbarara University Science and Technology (MUST). It has a total bed capacity of 350 beds, 20 of which are in the high-risk ward, where women with pregnancy-related complications, including hypertensive disorders of pregnancy, diabetes mellitus, heart disease, and coagulation disorders, are admitted. The facility conducts 9400 deliveries per year [12] with an average admission of 30 women with preeclampsia per month. Laboratory services to diagnose HELLP syndrome are also available at MRRH.

### Study population

The study population included all pregnant women with preeclampsia/eclampsia admitted to the high-risk ward of MRRH. The admission notes of patients admitted with a diagnosis of preeclampsia or eclampsia were checked to confirm the diagnosis as evidenced by a high blood pressure of systolic pressure ≥ 140mmHg and/or diastolic pressure ≥ 90mmHg, the presence of proteinuria and/or features of end-organ damage, including a history of eclamptic fits.

### Sample size and sampling

The sample size was calculated using OpenEpi version 3.01 [13]. For the descriptive objective aimed at determining the prevalence of HELLP syndrome. We assumed a 95% confidence interval, 5% margin of error, and accounting for a 10% nonresponse rate. We used a study conducted at MRRH, which reported a prevalence of 7.8% among women with hypertensive disorders during pregnancy [14]. The calculated sample size was 110 participants.

For the analytical objective that aimed to identify factors associated with HELLP syndrome, assumed power of 80%, percentage of unexposed with outcome of 24%, percentage of exposed with outcome of 39%, and odds ratio of 2, as obtained from a study conducted at Kenyatta National Hospital, Kenya; the exposure of interest was epigastric pain [15]. The calculated sample size was 324 participants. Considering that our setting admits approximately 30 women monthly with preeclampsia/eclampsia (180 women in 6 months), we adjusted the sample size for the finite population using the formula n1 = N*n/(n+(N-1)), where n1 represents the final sample size, N is the finite population, and n is the sample size before correction. The resulting adjusted sample size was 116. Factoring in a 10% non-response rate, the final sample size was adjusted to 129 participants with a diagnosis of preeclampsia/eclampsia.

### Data collection and laboratory procedures

All pregnant women admitted to the high-risk ward at MRRH were assessed for eligibility. The data were collected from eligible participants by trained research assistants using an interviewer-administered questionnaire. Venous blood (2 ml) was collected from the participants’ antecubital fossa on a nondominant arm into an EDTA-containing tube (purple top) vacutainer for complete blood count (CBC) using a hematology analyzer (SYMEX XN-550 Germany), and 4 ml of blood was collected into a red top vacutainer for lactate dehydrogenase (LDH) and aspartate amino transferase (AST) using an immunoassay machine (HUMASTER 200 Germany) at the MRRH Clinical Laboratory.

### Study variables

The outcome variable, complete HELLP syndrome, was defined based on the Tennessee classification as presence of all the following: aspartate aminotransferase (AST) ≥ 70 IU/L, lactate dehydrogenase (LDH) ≥ 600 IU/L, and a platelet count < 100,000 cells/µL [16].

The independent variables included age, parity, gestational age, admission blood pressure, education level, residence, referral status, history of headache, epigastric pain, previous history of preeclampsia, HIV serostatus, and history of intrauterine fetal death in previous pregnancy. Residing within town councils or municipalities was classified as urban, whereas living outside these administrative areas was categorized as rural. A woman was considered to have been referred if she had a formal referral letter or any documentation of referral in her medical records. HIV status was determined based on the client’s status in the three months preceding the interview, provided that the result was documented in the mother’s antenatal records. If there was no documented result, an HIV test was performed.

### Data management and analysis

The data were entered into REDCap electronic data capture tools hosted at the Mbarara University of Science and Technology (MUST) Department of Obstetrics and Gynecology and exported to STATA version 15 (StataCorp, College Station, Texas, USA) for analysis. The demographic, obstetric, medical, and clinical characteristics of the study participants are expressed as descriptive statistics, such as frequencies/percentages. The characteristics of participants with and without HELLP syndrome were compared. For categorical variables, we used the chi-square test or Fisher’s exact test; for continuous variables, we used Student’s t test.

The prevalence of HELLP syndrome was determined as the proportion of women with preeclampsia/eclampsia diagnosed with HELLP syndrome and expressed as a percentage. To determine the factors associated with HELLP syndrome, we performed univariable and multivariable modified Poisson regression analyses. We used a generalized linear model regression of the Poisson family with a log link and robust standard errors. We reported crude and adjusted prevalence ratios as the measures of association, along with their corresponding 95% confidence intervals. Variables with *p* < 0.2 in the univariate analysis or those that were biologically plausible to HELLP syndrome (e.g., gestational age, prior history of preeclampsia, creatinine level and gravidity) were included in the final multivariable model. The adjusted prevalence ratios and 95% confidence intervals at a significance level of *p* < 0.05 were reported.

## Results

**Fig. 1:**
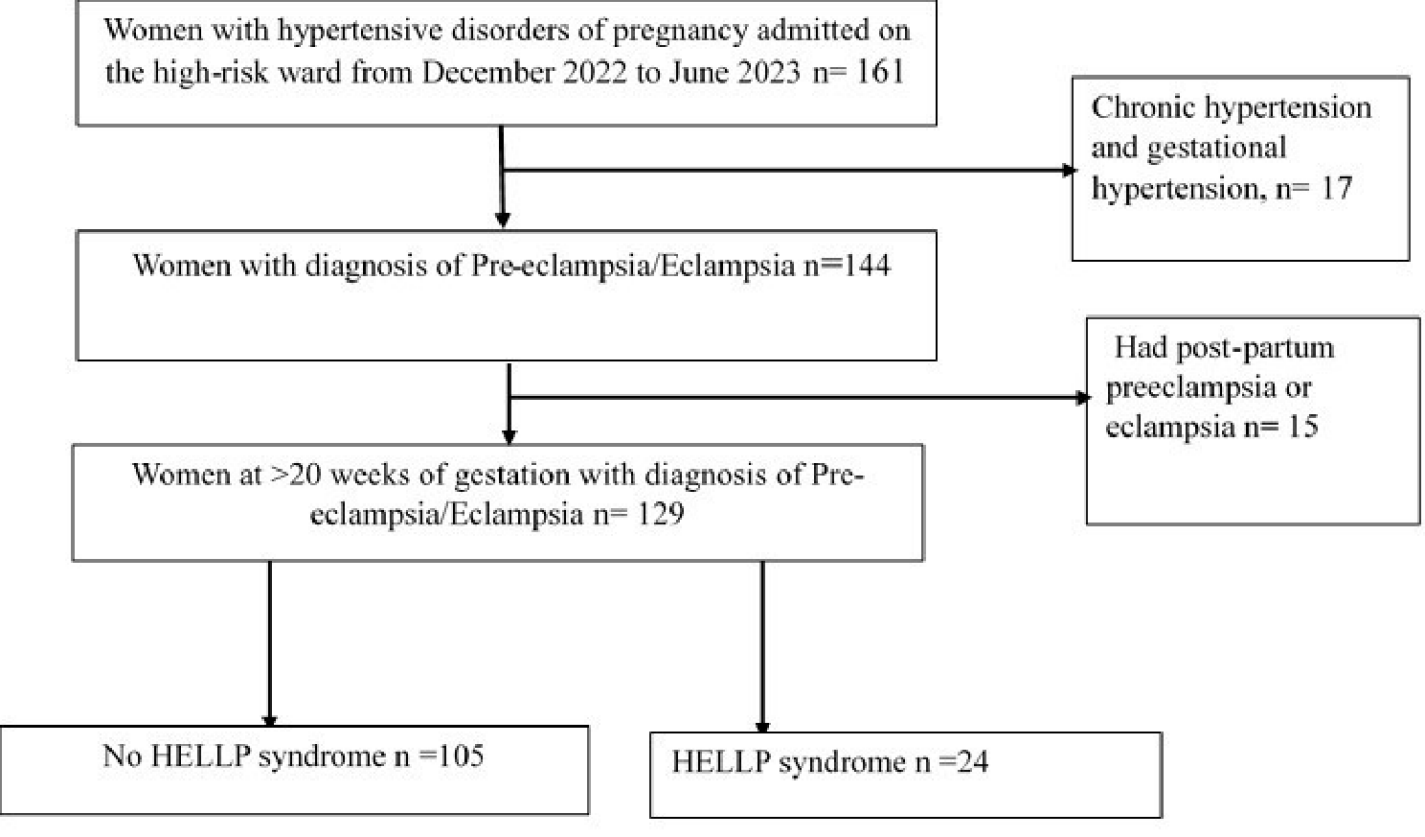
Flow chart for recruitment of participants at Mbarara Regional Referral Hospital, southwestern Uganda, December 2022–June 2023

Out of 161 women admitted to the high-risk ward with hypertension in pregnancy during the study period, 144 women diagnosed with preeclampsia/eclampsia at a gestational age greater than 20 weeks were screened for eligibility. Fifteen [15] participants who had postpartum preeclampsia or eclampsia were not included in the study. Therefore, 129 participants were enrolled in the study, as shown in Fig. 1.

### Sociodemographic characteristics of the study participants

The mean age was 28.3 ± 6.6 years with an age range of 15 to 45 years; the majority of participants were in the 20–34 years category (72.9%). The highest proportion were rural residents (55.0%), were married (74.4%) and had a primary level of education (34.1%). The distributions of sociodemographic characteristics were largely comparable between women with and without HELLP syndrome, with the exception of marital status. A significantly greater proportion (95.8%) of women with HELLP syndrome were married compared to 4.2% those who were not married (*p* = 0.008) (Table 1).

**Table 1:** Sociodemographic characteristics of the study participants

Characteristics			HELLP* Syndrome		
		Total (*N* = 129)	Yes (*n* = 24)	No (*n* = 105)	*P* value
		***n (%)***	***n (%)***	***n (%)***	
Age (years), mean (SD*)		28.3 ± 6.6	27.8 ± 7.2	28.4 ± 6.5	0.690
Maternal age category					0.670
	< 20	12 (9.3)	3 (12.5)	9 (8.6)	
	20–34	94 (72.9)	18 (75.0)	76 (72.4)	
	> 34	23 (17.8)	3 (12.5)	20 (19.0)	
Residence					0.200
	Urban area	58 (45.0)	8 (33.3)	50 (47.6)	
	Rural area	71 (55.0)	16 (66.7)	55 (52.4)	
Level of Education					0.260
	None	12 (9.3)	0 (0.0)	12 (11.4)	
	Primary	44 (34.1)	10 (41.7)	34 (32.4)	
	Secondary	39 (30.2)	6 (25.0)	33 (31.4)	
	Tertiary	34 (26.4)	8 (33.3)	26 (24.8)	
Marital status					0.008
	Not married	33 (25.6)	1 (4.2)	32 (30.5)	
	Married	96 (74.4)	23 (95.8)	73 (69.5)	
New partner					0.910
	No	101 (78.3)	19 (79.2)	82 (78.1)	
	Yes	28 (21.7)	5 (20.8)	23 (21.9)	

*HELLP Hemolysis Elevated liver enzymes low platelets; SD: Standard deviation

Among the 129 participants, the majority had pregnancies at a gestational age ≥ 34 weeks (61.2%), attended ≥ 4 ANC visits (58.1%), and had singleton pregnancies (93.8%). Approximately one-third of mothers (36.4%) had been referred from other facilities. Overall, most of the medical and obstetric characteristics of the participants with HELLP syndrome did not significantly differ from those without HELLP syndrome, except for HIV status. The proportion of women with HELLP syndrome living with HIV was 12.5%, compared to 33% among those without HELLP syndrome (*p* = 0.043) (Table 2).

**Table 2:** Medical and obstetric characteristics of participants

Characteristics			HELLP* Syndrome		
		Total (*N* = 129)	Yes (*n* = 24)	No (*n* = 105)	*P* value
		***n (%)***	***n (%)***	**n (%)**	
Gravidity					0.320
	Primigravida (1)	39 (30.2)	5 (20.8)	34 (32.4)	
	Multigravida (2–4)	68 (52.7)	16 (66.7)	52 (49.5)	
	Grand multigravida (≥ 5)	22 (17.1)	3 (12.5)	19 (18.1)	
Gestational age (weeks)					
	< 34	50 (38.8)	12 (50.0)	38 (36.2)	0.230
	≥ 34	79 (61.2)	12 (50.0)	67 (63.8)	
Number of ANC* visits					0.371
	< 4	54 (41.9)	12 (50.0)	42 (40.0)	
	≥ 4	75 (58.1)	12 (50.0)	63 (60.0)	
Type of pregnancy					0.652
	Singleton	121(93.8)	23 (95.8)	98 (93.3)	
	Multiple	8 (6.2)	1 (4.2)	7 (6.7)	
Referral status					0.078
	Yes	47 (36.4)	5 (20.8)	42 (40.0)	
	No	82 (63.6)	19 (79.2)	63 (60.0)	
History of preeclampsia		30 (23.3)	6 (25.0)	24 (22.9)	0.820
History of IUFD*		10 (7.8)	4 (16.7)	6 (5.7)	0.071
Malaria episode during this pregnancy		14 (10.9)	2 (8.3)	12 (11.4)	0.663
Diabetes mellitus		4 (3.1)	1 (4.2)	3 (2.9)	0.742
Chronic hypertension		14 (10.9)	3 (12.5)	11 (10.5)	0.771
HIV status					0.043
	HIV negative	91 (70.5)	21 (87.5)	70 (66.7)	
	HIV positive	38 (29.5)	3 (12.5)	35 (33.3)	
Family history of hypertension		30 (23.3)	7 (29.2)	23 (21.9)	0.454

*IUFD: Intrauterine fetal death; ANC: Antenatal care; HELLP: Hemolysis Elevated liver enzyme low platelets

### Clinical profile of the study participants

With regard to clinical symptoms, participants with HELLP syndrome demonstrated a significantly higher prevalence of epigastric pain (87.5%) than did those without HELLP syndrome (60.0%) (*p* = 0.011). However, there were no significant differences in the occurrence of headache, blurred vision, or convulsions between the two groups (Table 3).

With regard to clinical signs, participants with HELLP syndrome had higher systolic and diastolic blood pressure than did those without HELLP syndrome (*p* = 0.034 and *p* = 0.005, respectively). Participants with HELLP syndrome had a significantly greater mean creatinine level (1.9 vs. 1.3, *p* = 0.001). Mean hemoglobin and urea concentrations did not significantly differ between participants with and without HELLP syndrome (Table 3).

**Table 3:** Clinical and laboratory profile of the study participants

Characteristics			HELLP* Syndrome		
		Total (*n* = 129)	Yes (*n* = 24)	No (*n* = 105)	*P* value
		***n (%)***	***n (%)***	***n (%)***	
**Clinical symptoms**					
Epigastric pain		84 (65.1)	21 (87.5)	63 (60.0)	0.011
Headache		87 (67.4)	19 (79.2)	68 (64.8)	0.171
Blurred vision		55 (42.6)	11 (45.8)	44 (41.9)	0.732
Convulsions		11 (8.5)	3 (12.5)	8 (7.6)	0.061
**Clinical Signs**					
Admission BP* (mmHg)					0.460
	140/90 < 160/110	63 (48.84)	9 (37.50)	54 (51.43)	
	BP ≥ 160/110	66 (51.16)	15 (62.5)	51 (48.57)	
Systolic BP (mean ± SD*)		162.1 ± 23.8	171.42 ± 28.5	160.03 ± 22.2	0.034
Diastolic BP (mean ± SD)		103.7 ± 16.3)	111.96 ± 19.8	101.8 (14.8)	0.005
**Laboratory assessments**					
Proteinuria					0.240
	Nil	18 (14.0)	4 (16.7)	14 (13.3)	
	1+	14 (10.9)	0 (0.0)	14 (13.3)	
	2+	46 (35.7)	8 (33.3)	38 (36.2)	
	≥ 3+	51 (39.5)	12 (50.0)	39 (37.1)	
Creatinine (mg/dL) (mean ± SD)		1.4 ± 1.3	1.9 ± 1.4	1.3 ± 1.2	0.001
Urea (mg/dL) (mean ± SD)		54.9 ± 81.4	101.6 ± 171.9	44.1 ± 31.5)	0.100
Hemoglobin (g/dl) (mean ± SD)		11.9 ± 2.4)	11.1(± 3.2)	12.2 (± 2.2)	0.052

*HELLP: Hemolysis Elevated liver enzyme low platelets SD: standard deviation; BP: Blood pressure

### Prevalence of HELLP syndrome

Among the 129 pregnant women diagnosed with preeclampsia/eclampsia at MRRH, the proportion of women with elevated liver enzymes was 38.8% (50/129), low platelet counts 27.1% (35/129) and hemolysis 47.3% (61/129). The prevalence of complete HELLP syndrome was 18.6% (*n* = 24; 95% CI = 12.7–26.3%).

### Factors associated with HELLP syndrome

In the adjusted analysis, referral status, maternal age less than 20 years, and a history of epigastric pain emerged as independent factors associated with HELLP syndrome. Women who were referred were 3.1 times (aPR = 3.14, 95% CI = 1.27–7.72, *p* = 0.013) more likely to have HELLP syndrome than women who were not referred. Approximately 5-fold more women aged < 20 years had HELLP syndrome (aPR = 4.96, 95% CI = 1.57–15.65, *p* = 0.006) than did those aged between 20 and 34 years. Participants with a history of epigastric pain were 5.9 times (aPR = 5.89, 95% CI = 1.41–14.63, *p* = 0.015) more likely to have HELLP syndrome, as shown in Table 4.

**Table 4:** Factors associated with HELLP syndrome among patients with preeclampsia/eclampsia at Mbarara Regional Referral Hospital, southwestern Uganda

Variables			HELLP Syndrome		Unadjusted analysis		Multivariable analysis	
			Yes (*n* = 24)	No (*n* = 105)	cPR (95% CI)	*P* value	aPR (95% CI)	*P* value
			*n* (%)	*n* (%)				
Maternal age								
		< 20	3 (12.5)	9 (8.6)	1.31[0.45,3.80]	0.625	4.96[1.57,15.65]	0.006
		20–34	18 (75.0)	76 (72.4)	Ref.		Ref.	
		> 34	3 (12.5)	20 (19.0)	0.68[0.22,2.23]	0.509	0.32[0.90,1.11]	0.071
Marital status								
		Not married	1 (4.2)	32 (30.5)	0.13[0.18,0.91]	0.040	0.46[0.18,1.20]	0.111
		Married	23 (95.8)	73 (69.5)	Ref.		Ref.	
Referral status								
		No	5 (20.8)	42 (40.0)	Ref.		Ref.	
		Yes	19 (79.2)	63 (60.0)	2.18[0.87,5.47]	0.098	3.14[1.27,7.72]	0.013
Gravidity								
		I	5 (20.8)	34 (32.4)	0.54[0.22,1.38]	0.199	0.89[0.32,2.45]	0.817
		II-IV	16 (66.7)	52 (49.5)	Ref.		Ref.	
		≥V	3 (12.5)	19 (18.1)	0.54[0.22,1.81]	0.348	0.57[0.19,1.78]	0.336
Gestational age (weeks)								
	< 34 (Early)		12 (50.0)	38 (36.2)	1.58[0.77,3.25]	0.213	1.03[0.46,2.33]	0.944
	≥ 34 (Late)		12 (50.0)	67 (63.8)	Ref.		Ref.	
Prior preeclampsia								
		No	18 (75.0)	81 (77.1)	Ref.		Ref.	
		Yes	6 (25.0)	24 (22.9)	1.10[0.48,2.53]	0.822	0.88[0.38,2.04]	0.769
Chronic hypertension								
		No	21 (87.5)	94 (89.5)	Ref.		Ref.	
		Yes	3 (12.5)	11 (10.5)	1.17[0.39,3.45]	0.771	0.81[0.27,2.48]	0.718
History of convulsions								
		No	21 (87.05)	97 (92.4)	Ref.		Ref.	
		Yes	3 (12.5)	8 (7.6)	3.27[0.91,11.81]	0.070	2.68[0.71,10.05]	0.145
Epigastric pain								
		No	3 (12.5)	42 (40.0)	Ref.		Ref.	
		Yes	21 (87.5)	63 (60.0)	3.75[1.18,11.95]	0.025	5.89[1.41,14.63]	0.015
Headache								
		No	5 (20.8)	37 (35.2)	Ref.		Ref.	
		Yes	19 (79.2)	68 (64.8)	1.83 [0.73,4.59]	0.195	0.42[0.10,1.78]	0.238
Creatinine (mg/dl)								
		< 1.1	7 (29.2)	66 (62.9)	Ref.		Ref.	
		≥ 1.1	17 (70.8)	39 (37.1)	3.16 [1.41,7.12]	0.005	1.39[0.57,3.39]	0.472

cPR: crude prevalence ratio; aPR: adjusted prevalence ratio: CI: confidence interval

## Discussion

In this study, approximately 2 out of every 10 women diagnosed with preeclampsia or eclampsia at the MRRH in southwestern Uganda had HELLP syndrome. Referral from another health facility, being a teenage mother (maternal age < 20 years), and having a history of epigastric pain emerged as independent factors significantly associated with HELLP syndrome.

The prevalence in this study of 18.6% is similar to that found in Ethiopia, which had a prevalence of 13% [17]. The study from Ethiopia was a systematic review and meta-analysis of cross sectional studies that were conducted at tertiary hospitals in Ethiopia. In contrast, a study in Zimbabwe reported a lower prevalence of 9.1% [3]. In the study conducted in Zimbabwe, the diagnosis of HELLP syndrome was based on what was reported in the participant clinical notes, unlike in our study, where participants had laboratory tests conducted. The true burden of HELLP syndrome among women with preeclampsia could have been underestimated in the Zimbabwe study. Given the potential for under diagnosis of HELLP syndrome in the absence of a laboratory diagnosis, early detection of HELLP syndrome may necessitate integrating comprehensive diagnostic capabilities into maternal health services, particularly in resource-constrained settings like Uganda, to ensure prompt management of women with preeclampsia at risk HELLP syndrome.

In this study, teenage mothers (aged < 20 years) were 4.96 times more likely to have HELLP syndrome than were those in the older age group (20 to 35 years). However, studies performed in Canada and the United Kingdom showed that mothers who were 35 years or older were more likely to have HELLP syndrome [6, 7]. The differences in risk according to age groups between developed and developing countries may also reflect the differences in the mean age of pregnancy within these settings. These findings suggest that both younger (< 20 years) and older (≥ 35 years) mothers, could be at risk of maternal complications, including HELLP syndrome.

In this study, women who were referred from lower-level health facilities were 3 times more likely to have HELLP syndrome than were those who had not been referred. This finding is similar to what was found in a study in Taiwan, where women who were referred to a tertiary hospital had a greater likelihood of having HELLP syndrome [18]. Referral from lower-level facilities maybe due to severity of disease. Referral may also be associated with delayed diagnosis contributing to the progression of preeclampsia to severe states like HELLP syndrome as evidenced by a qualitative study in rural Bangladesh [19]. A prospective cohort study at MRRH revealed that women with preeclampsia who had been referred had more severe maternal and perinatal outcomes [14]. Early referral of women with preeclampsia to tertiary centers could facilitate timely initiation of preeclampsia management and mitigate progression of preeclampsia to severe states like HELLP syndrome. Nevertheless, further studies exploring referral pathways for women with preeclampsia/eclampsia in our setting are warranted to better inform policy programming and pre-referral interventions for this study population at risk of HELLP syndrome.

Women with preeclampsia or eclampsia and epigastric pain were five times more likely to have HELLP syndrome than were those without epigastric pain. Similar findings have been reported by other studies [11, 15]. In HELLP syndrome, fibrin and fatty acids are deposited in the intrahepatic sinusoids, causing hepatic sinusoidal blockage and hepatic congestion and necrosis with intraparenchymal and subscapular hemorrhages [20]. Epigastric pain follows the resultant stretching of the liver capsule and manifests as somatic pain at the epigastrium/right upper quadrant because visceral pain from afferent parasympathetic fibers from the liver is referred to dermatomes at the epigastrium [21]. The implication of this finding is that, in settings where laboratory services are limited, the presence of epigastric pain in women with preeclampsia or eclampsia may serve as a crucial clinical indicator of HELLP syndrome. Identifying and recognizing epigastric pain in conjunction with hypertensive disorders during pregnancy could guide clinical decision-making in resource-constrained environments, facilitating prompt interventions and appropriate management to mitigate the risks associated with HELLP syndrome.

The strength of our study lies in the comprehensive and objective diagnosis of HELLP syndrome using the three laboratory criteria. This study, however, has several limitations that should be taken into consideration when interpreting the study findings. First, as a single-center study that was conducted at a regional referral hospital in a low-resource setting in southwestern Uganda, the generalizability of the findings may be limited to healthcare settings within similar contexts in southwestern Uganda and sub-Saharan Africa. Second, we did not study women with postpartum preeclampsia or eclampsia and this may restrict the applicability of the findings to the broader population of women with preeclampsia or eclampsia, potentially overlooking crucial insights into the prevalence and factors associated with HELLP syndrome in the postpartum period. We recommend further investigations among postpartum women and maternal and neonatal outcomes among women who have preeclampsia and HELLP syndrome. Longitudinal studies should assess maternal and neonatal outcomes to provide a more comprehensive understanding of the impact of HELLP syndrome in resource-limited settings.

## Conclusions

Approximately 2 out of every 10 women diagnosed with preeclampsia or eclampsia at MRRH in southwestern Uganda had HELLP syndrome. HELLP syndrome was more common among teenage mothers, those with a history of epigastric pain and those referred from lower health facilities. Incorporating routine laboratory testing for HELLP syndrome in the diagnostic protocol for preeclampsia or eclampsia, especially among teenage mothers, those experiencing epigastric pain, and those referred from lower health facilities, could enhance timely detection and management of mothers with preeclampsia whose pregnancies are complicated by HELLP syndrome.

## Data Availability

The datasets used and/or analyzed during the current study are available from the corresponding author upon reasonable request.

## Abbreviations

AST: Aspartate transferase
BP: Blood pressure
CBC: Complete blood count
HELLP: Hemolysis Elevated Liver Enzymes, Low Platelet Count
LDH: Lactate dehydrogenase
MRRH: Mbarara Regional Referral Hospital
MUST: Mbarara University of Science and Technology
UNCST: Uganda National Council for Science and Technology
